# Targeting pre-mRNA splicing in cancers: roles, inhibitors, and therapeutic opportunities

**DOI:** 10.3389/fonc.2023.1152087

**Published:** 2023-06-05

**Authors:** Shinsuke Araki, Momoko Ohori, Masato Yugami

**Affiliations:** Research, Takeda Pharmaceutical Company Limited, Fujisawa, Kanagawa, Japan

**Keywords:** alternative splicing, splicing modulator, small molecule, SRPK, CLK, CDK12, patient stratification

## Abstract

Accumulating evidence has indicated that pre-mRNA splicing plays critical roles in a variety of physiological processes, including development of multiple diseases. In particular, alternative splicing is profoundly involved in cancer progression through abnormal expression or mutation of splicing factors. Small-molecule splicing modulators have recently attracted considerable attention as a novel class of cancer therapeutics, and several splicing modulators are currently being developed for the treatment of patients with various cancers and are in the clinical trial stage. Novel molecular mechanisms modulating alternative splicing have proven to be effective for treating cancer cells resistant to conventional anticancer drugs. Furthermore, molecular mechanism-based combination strategies and patient stratification strategies for cancer treatment targeting pre-mRNA splicing must be considered for cancer therapy in the future. This review summarizes recent progress in the relationship between druggable splicing-related molecules and cancer, highlights small-molecule splicing modulators, and discusses future perspectives of splicing modulation for personalized and combination therapies in cancer treatment.

## 1 Introduction

Alternative pre-mRNA splicing is a critical molecular mechanism underlying the generation of diverse proteins and plays a pivotal role in various biological processes, such as development, differentiation, growth, and apoptosis (1–3). Alternative splicing of RNA-binding protein FOX1 homologue 1 plays key roles in brain development and neuronal differentiation, as the nucleic and cytoplasmic isoforms regulate approximately 500 alternative spliced cassette exons that maintain neuron function or control mRNA expression of synaptic and autism-related genes (4). In heart development, alternative spliced isoforms of Titin control the elasticity of titin, thereby determining sarcomere length (4). Alternative splicing is evolutionarily conserved, but yet diverged, as evidenced by comparisons of organ transcriptomes from vertebrate species spanning approximately 350 million years of evolution (5–7). Recent studies have linked dysregulation of the components of alternative splicing machinery with various diseases, especially cancers. Analyses of >8,000 tumors across 32 types showed that tumors have up to 30% more alternative splicing events than normal samples (8). In contrast, thousands of splicing variants were not present in nonmalignant tissues (8), suggesting potential links between splicing abnormalities and cancer progression and metastasis. Alternatively spliced isoforms associated with cancer have been implicated in tumor progression, migration, apoptosis, and angiogenesis. Several spliced isoforms, including Bcl-xL and MCL1L, are up-regulated in tumor cells as anti-apoptotic factors (3). In addition, spliced isoforms are responsible for resistance against anticancer therapies, such as androgen receptor (AR)-targeted therapy for prostate cancer (9). The aberrant isoform of AR-V7 is constitutively active, thereby contributing to the development and progression of castration-resistant prostate cancer and resistance to AR-targeted therapy (10).

Multiple strategies have been implemented to target tumor cells, including small-molecule compounds and antisense oligonucleotides. To target alternative splicing machinery is promising candidates for cancer treatment, and multiple molecules including kinases are involved in pre-mRNA splicing events in cancer (2, 11). In this review, we highlight the druggable splicing-related molecules and the newly developed compounds to target alternative splicing for cancer therapy. First, we introduce the pre-mRNA splicing machinery and mutations of splicing-related genes in cancer. Then, we discuss why targeting splicing molecules is effective for cancer treatment. Next, we describe the dysregulation of splicing-related proteins and small-molecule compounds in cancer. Finally, we discuss the therapeutic potential of splicing modulators for cancer treatment from the perspective of patient stratification and drug combination strategy.

## 2 The spliceosome machinery

**Figure 1:**
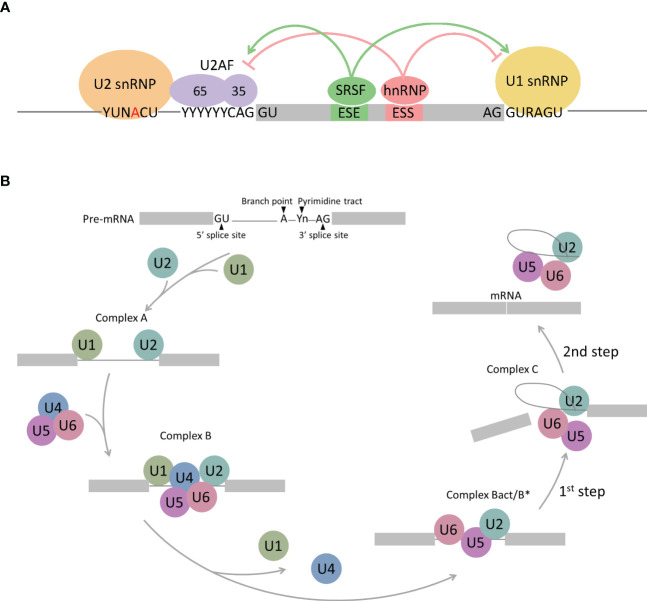
Schematic representation of spliceosome assembly. **(A)** Complex E regulated by RNA-binding proteins. SR proteins (SRSFs) bind to Exonic splicing enhancers (ESE), thereby stimulating splicing at up- and downstream splice sites by facilitating the interaction of U2AF or the U1 snRNP with the RNA. The hnRNP proteins bind to Exonic splicing silencers (ESS), subsequently suppress splicing at up- and downstream splice sites by antagonizing SR proteins. **(B)** Spliceosome assembly pathway. 5′ splice site is recognized by U1 snRNP, followed by U2 snRNP binding to the branch point (complex A). U4/U6/U5 tri-snRNP complex binds to complex A to form complex B and conformational rearrangements releases U1 and U4 snRNP, which results in activation of the spliceosome (complex B act/B*). Further conformational rearrangements and changes in protein composition catalyzes the first step of the splicing reaction, leading to the formation of a lariat intermediate (complex C). An additional conformational switch leads to the second catalytic step, rendering the spliced product (mRNA) and the intron lariat.

**Figure 2:**
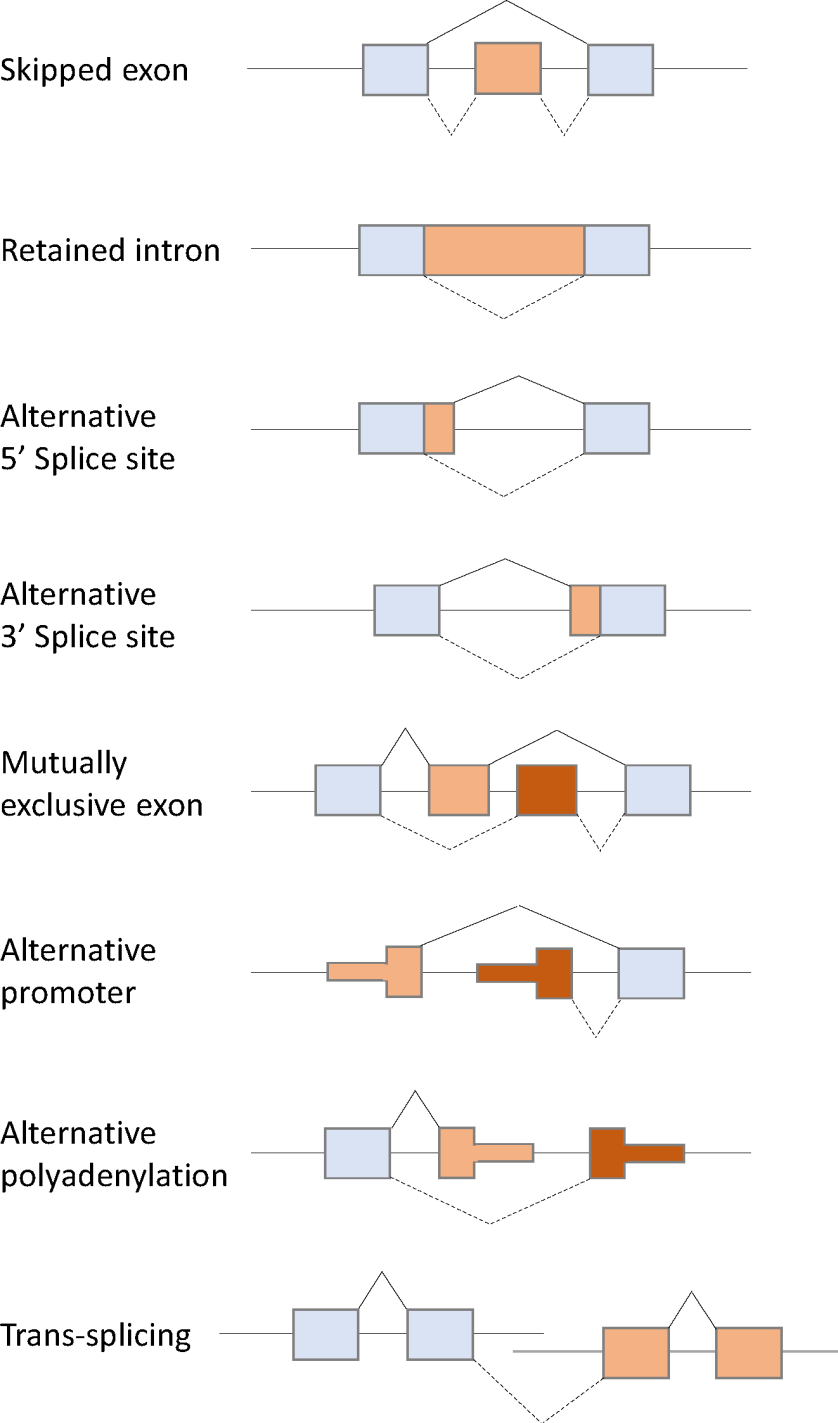
Schematic diagram of eight types of alternative splicing: Exon skipping, intron retention, alternative 5′ splice site, alternative 3′ splice site, mutually exclusive exon, alternative promoter, alternative polyadenylation, and trans-splicing.

Pre-mRNA splicing is performed by the spliceosome, which comprises multicomponent five ribonucleoprotein complexes (snRNPs; U1, U2, U4, U5, and U6), each composed of specific small nuclear RNAs (snRNAs), a number of associated proteins, and >200 additional polypeptides not directly bound to snRNPs (12–14). The spliceosome is assembled and activated through a series of ATP/GTP-dependent reactions from complex E to complexes A, B, and C *via* RNA–RNA and RNA–protein interactions. Spliceosome assembly starts with the recognition of the 5′ splice site (5′ss) by U1 snRNP *via* base paring interactions with U1 snRNA and the 5′-end of the intron. Subsequently, U2AF1, U2AF2, and SF1 interact with the 3′ss region, including the polypyrimidine tract and the AG dinucleotide, and lead to the formation of complex E (**Figure 1A**). The next step in spliceosome assembly involves ATP-dependent stable binding of U2 snRNP at the branch site through base pairing interactions with U2 snRNA, thereby leading to the formation of complex A (**Figure 1B**). SF3B1 is one of the key components of U2 snRNP that recognizes the branch site and the U2 snRNA–pre-mRNA helix and facilitates the approximation of the branch-site adenosine to the 5′ss. Other components of complex A, such as RBM10, RBM39, and RBM15, modulate 3′ss recognition, and RBM15 is regulated by protein arginine methyltransferase 1 (PRMT1). Recognition of splice sites by U1 and U2 snRNPs is assisted and modulated by several other RNA-binding proteins, such as arginine–serine-rich (SR) proteins, heterogeneous nuclear ribonucleoproteins (hnRNPs), and RNA-binding motif proteins. SR proteins, such as SRSF1 and SRSF2, bind to an exonic splicing enhancer (ESE) or intronic splicing enhancer (ISE) to promote exon selection by recruiting spliceosomal components, U1 or U2 snRNPs. After initial recognition of the splice sites by U1 and U2 snRNPs, subsequent recruitment of the U4/U6-U5 tri-snRNP complex to form complex B leads to the formation of the catalytically active conformations of the spliceosome. Then, pre-mRNA intron removal proceeds with two sequential transesterification reactions that are initiated by the nucleophilic attack of the 5′ss by the branch-site nucleotide, resulting in the formation of an intro lariat. The lariat is subsequently removed by 5′ss-mediated attack on 3′ss, producing a mature mRNA followed by spliceosome disassembly. Alternative splicing can arise from machineries, including exon skipping, intron retention, alternative 5′ splice site, alternative 3′ splice site, mutually exclusive exon, alternative promoter, alternative polyadenylation, and trans-splicing (**Figure 2**) (15–17).

## 3 Mutation of splicing factors

A recent whole genome sequence analysis showed that spliceosome-related genes are mutated in multiple tumors. The most commonly mutated splicing factor was SF3B1, which is a core component of U2 snRNP (18, 19). SF3B1 mutation was observed in 90% of patients with myelodysplastic syndrome (MDS) with ring sideroblasts, which is characterized by anemia, iron-rich mitochondria surrounding the nuclei of erythroid precursors, and a favorable prognosis (18–21). SF3B1 is mutated in the early stage of myeloid malignancies, whereas SF3B1 mutations in chronic lymphocytic leukemia (CLL) are most commonly subclonal and enriched in more advanced and aggressive disease (22, 23). SF3B1 mutations induce cryptic intron-proximal 3′ss selection through usage of a different branch point, since U2 snRNPs recognize the branch point sequence within the intron (24, 25). The most common mutation in SF3B1 is the K700E mutation, which is observed in myeloid malignancies, CLL, and multiple solid tumors. The SF3B1-K700E mutation has been implicated in cancer progression (26, 27). A conditional knock-in mouse with heterozygous expression of SF3B1-K700E causes progressive macrocytic anemia (27), and a knock-in mice with hematopoietic-specific expression of SF3B1-K700E impaired erythropoiesis and progressive anemia without ringed sideroblasts, accompanied by a reduction in hematopoietic stem cells (26). Additional hot spot mutations with SF3B1, such as R625 and E902, are enriched in melanomas and bladder carcinomas; however, the lineage specificity of these hotspot mutations remains to be elucidated (28). In multiple cancers, the SF3B1 mutation has been linked to dysregulation of several cellular pathways and functions, DNA damage response, R-loop formation, telomere maintenance, and Notch signaling (29–31). In addition, SRSF2, U2AF1, and ZRSR2 mutations are observed. SRSF2, one of the SR proteins, is frequently mutated in acute myeloid leukemia (AML) (32). Canonical SRSF2 equally binds CCNG and GGNG sequences and induces alternative splicing (33). However, SRSF2 mutations preferably alter splicing of exons with C-rich sequences than with G-rich sequences (32, 34, 35). The SRSF2 mutation leads to splicing alterations of *EZH2*, which results in reduced expression by nonsense-mediated decay, thereby impairing hematopoietic differentiation *in vivo* (34). Additionally, frequent overlap of SRSF2 and IDH2 mutations alters splicing of INTS3, leading to leukaemogenesis due to reduced expression with nonsense-mediated decay (32). Hotspot mutations in U2AF1 are mainly located in the S34 or Q157 residue. S34 mutations promote inclusion of exons whose 3′ss is C-rich, whereas Q157 mutations enhance inclusion of exons whose 3′ss is G-rich. The U2AF1 mutation alters hematopoiesis and splicing in hematopoietic progenitor cells, thereby contributing to abnormal hematopoiesis *in vivo* (36). Mutations in ZRSR2 are identified in approximately 10% of patients with MDS (37, 38). These mutations are located sporadically across the coding regions, suggesting that these mutations are loss-of-function mutations and distinct from the change-of-function mutations in SF3B1, SRSF2, and U2AF1. ZRSR2 mutations, which predominantly occur in males, induce abnormal splicing *via* U12-dependent introns, leading to hematopoietic stem cell self-renewal and disorders (39, 40).

## 4 Druggable splicing-related proteins

### 4.1 Splicing-related protein kinases (SRPKs/CLKs)

**Figure 3:**
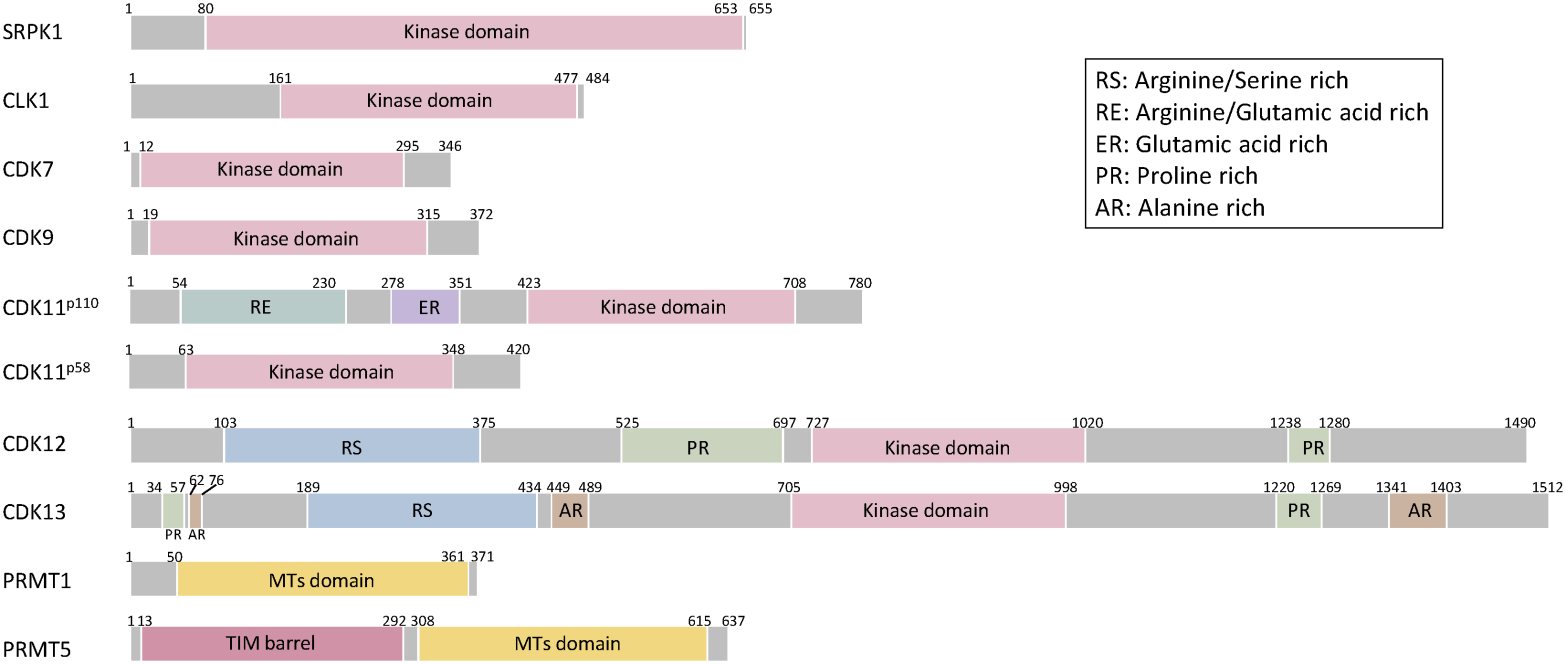
Schematic structures of splicing-related proteins. SRPK1, CLK1, CDK7, CDK9, CDK11, CDK12, CDK13, PRMT1, PRMT5. The Kinase domain of SRPK1 includes internal spacer sequence that separates the bipartite kinase catalytic core. CLK1 has a kinase domain at the C-terminus. CDK family proteins have consensus serine/threonine kinase domain. In addition to consensus kinase domain, CDK11 has two separate domains and arginine/glutamic acid rich (RE) domains are linked to association with RNA processing factors and glutamic acid rich (ER) domain support RE domain function and aide in keeping these proteins subnuclear. CDK11^p58^ is a short isoform of CDK11^p110^ missing 1-360 amino acids. CDK12 and CDK13 have additional arginine/serine rich (RS) domain, serve as docking sites for assembly of splicing factors and regulation of splicing and proline-rich (PR) domains serve as binding sites for Src-homoogy3, WW or profilin-domain-containing proteins. CDK13 has three alanine-rich (AR) domains. The methyl transferases domain of PRMT1 and 5 is consists of the catalytic Rossman fold and Β-barrel. PRMT5 adopts a TIM barrel structure at the N-terminal, which interacts with the C-terminal catalytic domains of adjacent monomers.

Members of kinase families, including serine–arginine protein kinases (SRPKs) and CDC-like kinases (CLKs), phosphorylate the RS domains of SR proteins, thereby regulating their subcellular localizations and interactions with ESEs or ISEs of pre-mRNAs. SRPKs (SRPK1, SRPK2, and SRPK3) are serine–threonine kinases that phosphorylate serine residues within serine/arginine (S/R) dipeptides enriched in SR proteins (41, 42). SRPKs contain a nonhomologous spacer insert domain that bifurcates the kinase domain and anchors the kinase in the cytoplasm through interactions with chaperones (43–45) (**Figure 3**). SRPKs phosphorylate SR proteins in the cytoplasm and induce their nuclear import by increasing their affinity to the transportin complex (46). Activated Akt induces SRPK autophosphorylation that switches the chaperones complex of SRPK, thereby enhancing SRPK nuclear translocation (45, 47). SRPKs and CLKs simultaneously regulate the shuttling of SR proteins between nuclear speckles and the nucleus *via* phosphorylation of different regions of SR dipeptides (48, 49). SRPK1 and SRPK2 are ubiquitously expressed in human tissues, whereas SRPK3 is predominantly expressed in muscle tissues (50, 51). Consistent with the expression pattern in humans, Srpk1-deficient mice show embryonic lethal and Srpk3-deficient mice show a type 2-specific myopathy (51, 52).

CLKs comprise four members, i.e., CLK1–4, and a phylogenetic kinome analysis revealed that CLK1 and CLK4, CLK2 and CLK3 have higher similarity (53). In addition to the phosphorylation of arginine/serine dipeptides, CLKs phosphorylate the SR proteins of serine/proline; this step is critical for binding of the SR protein by CLK1 but not for SRPK1-dependent reaction (54). CLKs have a C-terminal kinase domain and an N-terminal noncatalytic domain with a serine/arginine-rich region (55) (**Figure 3**). CLKs phosphorylate SR proteins in the nucleus. Subsequently, the kinase domain of SRPK1 interacts with the N-terminal domain of CLK1 and leads to the release of SR proteins from nuclear speckles and induces the interaction of SR proteins with U1 snRNP to proceed splicing machinery (56, 57). CLKs are ubiquitously expressed in human tissues and exhibit alternative splicing-independent functions. CLK2 phosphorylates the SR domain on peroxisome proliferator-activated receptor γ coactivator (PGC-1α) and functions as an insulin-regulated suppressor of hepatic gluconeogenesis through disruption of PGC-1α (58, 59). CLK4 phosphorylates nexilin and regulates cardiac function (60). Cardiac-specific Clk4-knockout mice show pathological myocardial hypertrophy with progressive left ventricular systolic dysfunction and heart dilation (60).

### 4.2 Cyclin-dependent kinases (CDK7/CDK9/CDK11/CDK12/CDK13)

Cyclin-dependent kinases (CDKs) are classified into cell cycle-related and transcriptional-related subfamilies based on their substrate specificity (42, 61). Among the transcriptional-related CDKs, CDK7, CDK9, CDK11, CDK12, and CDK13 are directly or indirectly involved in the regulation of alternative splicing through reversible phosphorylation of the C-terminal domain (CTD) of RNA polymerase II (pol II). CDK7 preferentially phosphorylates Ser5 and Ser7 of RNA Pol II CTD and regulates transcriptional initiation and alternative splicing (62, 63). In addition to the phosphorylation of pol II, CDK7 phosphorylates multiple splicing factors, such as SF3B1, U2AF2, CDK9, CDK12 and CDK13 (63). Thus, a CDK7-selective inhibitor alters multiple splicing events, such as exon skipping and retained intron type of splicing (63). CDK9 preferentially phosphorylates Ser2 of RNA Pol II CTD and controls pol II pause release, elongation, alternative splicing, and polyadenylation (64–66). CDK9, together with cyclin T, forms positive transcription elongation factor b (P-TEFb), which controls the elongation phase of transcription by RNA pol II. CDK9 inhibition abrogates polyadenylation and causes an elongation defect starting at the last exon of a protein-coding gene. SILAC phospho-proteomics analysis identified that multiple splicing factors, such as SF3B1, are phosphorylated by CDK7, and CDK9 inhibition promotes the loss of interaction of SF3B1 (66). CDK9 and PP2A regulate mRNA cleavage and polyadenylation as well as alternative poly(A) site usage, since inhibition of PP2A abrogates the effect of CDK9 inhibition on transcription at the 3′end of the gene (66). CDK11 is encoded by *CDK11A* and *CDK11B*, whose mRNA is translated into two major CDK11 proteins, CDK11^p110^ and CDK11^p58^ (61, 67). CDK11–cyclin L complex involves transcription and RNA processing, in particular, alternative splicing (68). In addition to preferential phosphorylation of Ser2 of RNA Pol II CTD, CDK11 phosphorylates threonine residues of SF3B1 at its N terminus during spliceosome activation, thereby promoting binding between SF3B1 and U5/U6 snRNAs (69).

CDK12 and CDK13 exhibit 92% similarity in their kinase homology domain; thus, these are regarded as structurally and functionally redundant kinases (70). CDK12/CDK13 preferentially phosphorylate Ser2 of RNA Pol II CTD and regulate pol II elongation, termination, and alternative polyadenylation (71–75). CDK12/CDK13 bind to cyclin K and active complex while regulating transcriptional elongation and preferentially express DNA damage response and repair (DDR) genes to protect cells from genomic instability. Inhibition of CDK12 using siRNAs or small-molecule compounds leads to a predominant decrease in the expression of long genes with high numbers of exons, including breast and ovarian cancer type 1 susceptibility protein 1 (*BRCA1*), ataxia telangiectasia and Rad3-related (*ATR*), FA complementation group I (*FANCI*), and FA complementation group D2 (*FANCD2*), thereby inducing spontaneous DNA damage (76). CDK12 loss leads to elongation defects and increases intronic polyadenylation of DDR genes depending on gene length and the U1 snRNP/polyadenylation signal ratio, since the U1 snRNP complex prevents premature termination *via* recognition and inhibition of cryptic poly(A) sites (72, 74, 77–79). Interestingly, SILAC analysis also identified that CDK12 phosphorylates SF3B1, similar to CDK7 and CDK9. CDK12 loss of function induces minimal effect on alternative splicing (74). The phosphorylation site of SF3B1 could be critical for the regulation of alternative splicing and polyadenylation. More detailed analysis of the phosphorylation of mRNA processing-related proteins is expected to be performed in the future.

### 4.3 PRMTs (PRMT1/PRMT5)

Protein arginine methyltransferase 5 (PRMT5) and 1 (PRMT1) are directly and indirectly involved in the splicing machinery, respectively. PRMT5 is an essential type II arginine methyltransferase and catalyzes the formation of symmetric demethylated arginine on a variety of proteins and histones, thereby regulating chromatin structure, gene transcription, cellular differentiation, and pre-mRNA splicing (80). Methylation by PRMT5 is required for recruiting substrate adaptors, such as spliceosome subunits, SmB/B′, SmD1, and SmD2, through a TIM barrel domain at the N terminus, thereby regulating mRNA processing, including splicing activity (81). PRMT1 is a type I arginine methyltransferase and catalyzes the formation of asymmetrically demethylated arginine (82). PRMT1 methylates the RNA-binding protein RBM15 that preferentially binds to the specific intron region of SF3b, thereby affecting alternative splicing (80). Regarding the structure, PRMT5 and PRMT1 have a conserved core including a Rossman fold for cofactor binding and a Β-barrel for substrate binding (83) (**Figure 3**). PRMT5 also has a triose-phosphate isomerase (TIM) barrel at the N terminus, which is required for enhanced methyltransferase activity by forming a complex with methylosome protein 50 (83, 84).

## 5 Dysregulation of druggable splicing-related proteins in cancer

### 5.1 Dysregulation of SRPKs in cancer

**Figure 4:**
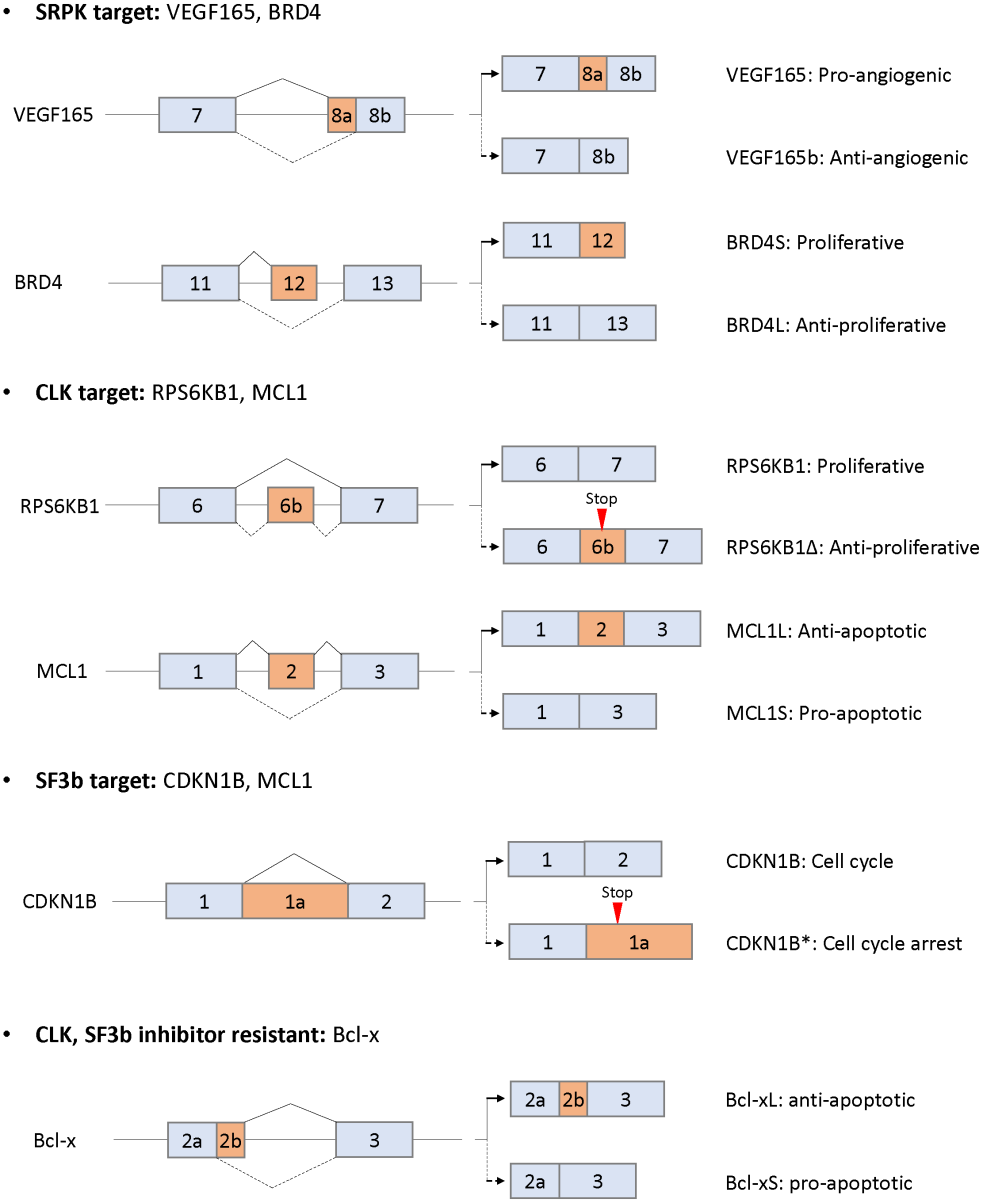
Alternative splicing as downstream targets for SRPK, CLK, and Sf3b and resistance to CLK or SF3b inhibitors. Only the alternatively spliced isoforms and the flanking exons are shown (not at scale). The corresponding isoforms are indicated by solid or dotted lines with the respective functions.

SRPK1 is upregulated in pancreatic, breast, colonic, prostate, and non-small cell lung carcinomas and leukemia (85–90). Upregulation of SRPK is correlated with poor prognosis of breast cancer metastasis-free survival and overall survival time of non-small cell lung carcinoma (91, 92). In contrast, upregulation of SRPK1 is observed in patients with germ cell tumors responding to standard chemotherapy with statistical significance (93). In addition, another study showed that reduced SRPK1 protein expression is associated with decreased response to chemotherapy in retinoblastoma patients (94). Interestingly, aberrant SRPK1 expression in either direction, up or down, is tumorigenic (52). Downregulated SRPK1 leads to constitutive Akt activation through impairing the recruitment of an Akt phosphatase, a pleckstrin homology domain leucine-rich repeat protein phosphatase (PHLPP1). Overexpression of SRPK1 also activates Akt by isolating PHLPP1. These results suggest that upregulated or downregulated expression of SRPK1 is observed in human tumors and that SRPK1 is involved in tumor progression, migration, and angiogenesis *via* splicing-dependent and -independent machineries. In splicing-dependent machinery, SRPK1 regulates multiple splicing and leads to tumor progression. In particular, the splicing variant of the vascular endothelial growth factor (*VEGF*) is dominantly regulated by SRPK1 (95). A spliced isoform, VEGF165, exhibits proangiogenic activity, whereas VEGF165b exhibits antiangiogenic activity (96). Aberrant upregulation of SRPK1 induces phosphorylation and nuclear translocation of SRSF1 and leads to splicing of VEGF165 in Wilms Tumor and melanoma (95, 97). Knockdown or chemical inhibition of SRPK1 alters splicing from VEGF165 to VEGF165b, thereby inhibiting angiogenesis and tumor progression (**Figure 4**). CRISPR-Cas9 screen shows that SRPK1 involves genetic vulnerability in AML cells (98). Comprehensive RNA-seq alternative splicing analysis using an SRPK inhibitor revealed that multiple events of splicing, especially exon skipping events, were altered in leukemic cells (90). SPHINX31, an SRPK inhibitor, suppressed growth and induced differentiation of human AML cells without causing any severe side effects, including hematopoiesis (90). Among altered splicing events, the bromodomain 4 (BRD4) isoform was altered from the short to the long isoform, eliminating BRD4 recruitment to chromatin involved in leukemogenesis including *BCL2* and *MYC* (**Figure 4**). These results suggest that SRPK plays a key role in tumor progression in terms of splicing regulation.

### 5.2 Dysregulation of CLKs in cancer

CLK1 and CLK2 are upregulated in breast and colorectal carcinomas and glioblastoma (99, 100). Upregulated expression of CLK1 and CLK2 is negatively correlated with prognosis in patients with kidney tumors and glioblastoma/colorectal carcinoma, respectively (100–102). The expression of CLK1 is regulated *via* the ubiquitin degradation mechanism during cell cycle (101). In the G2/M phase, the protein level of CLK1 is increased, whereas the protein levels of CLK2, SRPK1, and SR remain constant. Interestingly, thousands of cell cycle-dependent alternative splicing changes, including changes in the levels of RNA-binding proteins, are regulated by CLK1, suggesting that RNA metabolism is regulated by cell cycle and is aberrant in patients with tumors of higher mitotic index. Downregulation of CLKs inhibits cell growth and induces apoptosis in breast cancer and glioblastoma *in vitro* and *in vivo* (99, 102). CLKs also exhibit cancer cell growth inhibitory activity by modulating alternative splicing in multiple solid cancers (17, 103–107). A benzothiazole compound TG003 was first identified as a selective CLK inhibitor (108). TG003 inhibited cell proliferation and induced apoptosis in prostate cancer cells *in vitro* and *in vivo* through splicing changes of cancer-associated genes, including *CENPE*, *ESCO2*, *CKAP2*, *MELK*, *ASPH*, and *CD164* (107). TG003 also switched splicing to the short isoform of estrogen-related receptor Β, which is involved in G2/M cell cycle arrest and apoptosis induction (104). In addition, several compounds (Cpd-1, Cpd-2, and Cpd-3) with CLK inhibitory activity altered multiple splicing including cancer growth and survival-related genes, such as *RPS6KB1*, *EGFR*, *EIF3D*, and *PARP* (**Figure 4**) (105). The splicing alteration levels were correlated with cell growth inhibitory activity. Other CLK selective inhibitors, T3 and T-025, preferentially induced exon skipping type of splicing in colorectal cancer cell lines and other multiple cancer cell lines, since phosphorylated SR proteins play a central role in exon recognition (17, 103). Interestingly, T3 induces multiple conjoined genes, which arise from the upstream transcript to the downstream transcript of partner genes with poly(A) sites (17, 109, 110), whereas there is no evidence that conjoined genes are involved in cancer progression. Oral administration of T-025 at a concentration of 50 mg/kg twice daily/2 days per week for 3 weeks showed significant antitumor efficacy in MDA-MB-468 xenograft model without body weight loss (103). These results suggest that CLKs play central roles in cancer progression through predominant exon skipping of multiple genes in splicing alteration.

### 5.3 Dysregulation of CDKs in cancer

Multiple studies indicating aberrant expression of CDK7 in cancer have been reported (111). CDK7 expression is correlated with poor prognosis and overall survival in triple-negative breast cancer, gastric cancer, ovarian cancer, oral squamous cell carcinoma, hepatocellular carcinoma, and glioblastoma (112–118). Similar to CDK7, the expression of CDK9 has gained attention as a prognostic biomarker in bladder cancer, papillary thyroid carcinoma, pancreatic cancer, osteosarcoma, and breast cancer (119–124). CDK11 has emerged as a target of interest for cancer therapy (67, 125). Downregulation of CDK11 expression effectively inhibits cell proliferation and induces cell death in breast cancer, osteosarcoma, ovarian cancer, and liposarcoma cells as well as xenograft tumors (126–130). Upregulation of CDK11 protein expression levels are associated with poorer patient survival in osteosarcoma and ovarian cancer (127, 130). CDK12 is involved in cancer dysregulation *via* both gain of function with gene amplification and loss of function with mutations encoding the CDK12 locus. CDK12 is coamplified with the neighboring Her2 gene at the RNA, protein, and phosphosite levels in breast cancer (131). CDK12 is the most common coamplification gene, along with Her2, in breast, stomach, biliary tract, and colorectal cancers (132). Amplified CDK12 is associated with disease recurrence and poor prognosis and exhibits anti-HER2 therapy resistance in patients with breast cancer (133). Since CDK12 regulates the expression of genes involved in the activation of ErbB–PI3K–AKT or Wnt signaling cascades, CDK12 inhibition is more sensitive to antitumor activity in CDK12-Her2 coamplified cells than in CDK12 no-amplified Her2 positive cells *in vitro* and *in vivo* (133). Furthermore, since CDK12 predominantly regulates the expression of DDR-related genes, CDK12 inhibitors in combination with PARP inhibitors induce synthetic lethality in cancer cells (72, 134). In contrast to genomic amplification, CDK12 mutation or deletion has been reported in <5% of prostate and ovarian adenocarcinoma cases (135, 136). CDK12 mutations/deletion are loss-of-function type by nonsense mutations or loss-of-function type by frameshift mutation due to indels and are consistently associated with a particular genomic instability pattern induced by hundreds of tandem duplications in ovarian cancer and metastatic castration-resistant prostate cancer (136, 137). Regardless of the genomic instability pattern, the expression of *BRCA1*, *BRCA2*, or other genes encoding long transcripts was not affected under the CDK12-mutation condition (137), suggesting that different genomic instability pathways exist between CDK12 mutation and amplification status.

### 5.4 Dysregulation of PRMT1/PRMT5 in cancer

Upregulation of PRMT5 is observed in lymphomas, breast cancer, lung cancer, colorectal cancer, and glioblastoma (138–143). The expression level of PRMT5 is associated with poor overall survival (144). Involvement of PRMT5 in glioblastoma cell growth was identified by shRNA screen (145). Disruption of PRMT5 induces apoptosis and inclusion of detained introns, which are spliced and polyadenylated transcripts. In contrast to retained introns, detained introns remain in the nucleus and are finally post-transcriptionally spliced or degraded, thereby affecting the level of mature coding mRNA (145). A PRMT5 inhibitor (GSK591 or LLY-283) inhibited the growth of patient-derived glioblastoma stem cells in accordance with splicing alteration levels (146). PRMT inhibition altered multiple splicing events, particularly encoding cell cycle-related genes. In addition, the PRMT5 inhibitor induced antitumor effects in glioblastoma patient-derived xenografts (146). Orally available PRMT5 inhibitor (EPZ015666 [GSK3235025]) inhibits SmD3 methylation and RNA splicing as well as induces antitumor efficacy in mantle cell lymphoma (MCL) cells *in vitro* and *in vivo* (147). In addition, PRMT5 silencing or inhibition (EPZ015666) induced antitumor effect in glioblastoma cells *in vitro* and in an *in vivo* PDX model by removing detained introns, thereby regulating rapid expression of transcripts associated with proliferation (145).

## 6 Small-molecule splicing modulators for potential clinical cancer therapeutics

Multiple small-molecule compounds targeting splicing have been developed, and clinical trials have been conducted in patients with a variety of solid and hematopoietic tumors using multiple compounds. Currently, no approved compound modulating pre-mRNA splicing is available for cancer treatment. This section highlights promising small-molecule splicing modulators that can serve as anticancer therapeutics, especially those under clinical trials.

### 6.1 SF3b inhibitor

Compounds derived from natural products modulating pre-mRNA splicing have been identified as FR901464, spliceostatin A (SSA; a methyl ketal derivative of FR901464), sudemycin, pladienolide B, and E7107 (a 7-urethane derivative of plaedienolide D). These compounds bind to the SF3b subcomplex of U2 snRNP and modulate alternative splicing, thereby inhibiting cancer cell growth (148–150). Among SF3b complex binders, the detailed mechanism of action of spliceostatin A has been most clarified (149, 151–156). SSA produces the C-terminal spliced truncated form of p27 encoding *CDKN1B* mRNA *via* splicing modulation and stabilization of *CDKN1B* mRNA, which are the causes of G1 phase cell cycle arrest by treatment with spliceostatin A (**Figure 4**) (149, 151, 154). In addition, SSA downregulates the mRNA of *CCNE1*, *CCNE2*, and *E2F1*, which are additional genes involved in G1 arrest (152). One of the mechanisms downregulating gene expression is that SSA induces early dissociation of RNA polymerase II and decreases phosphorylation of the Ser2 of chromatin-bound RNA polymerase II (153). Other analysis showed that SSA predominantly induces intron retention type of splicing, the part of which leaks into the cytoplasm depending on the strength of the 5′ss and the length of the transcripts (156). SSA also affects the transcription of a nuclear long noncoding RNAs, including MALAT1. MALAT1 is prematurely cleaved and polyadenylated, and the truncated transcripts are exported into the cytoplasm and translated as aberrant proteins (155). Through this mechanism, the SF3b inhibitor, E7107, exerts antitumor activity in a human xenograft model without severe toxicity (157). Phase I clinical trials using E7107 in patients with solid tumors have been conducted. Intravenous treatment for 3 consecutive weeks showed that one patient had bilateral optic neuritis (158). Another administration on days 1 and 8 every 21 days revealed that two patients had visual loss due to optic nerve dysfunction at cycles 2 and 7 (159). The incidence of two cases of vision loss probably related to E7107 led to study discontinuation, although tumors in some patients responded partially to E7107. An orally available SF3b inhibitor, H3B-8800, preferentially inhibited tumor growth in spliceosome-mutant cancer cells, such as SF3B1 K700E, *in vitro* and *in vivo* by predominantly binding with short GC-rich regions (160). Currently, phase I clinical trial for H3B-8800 is ongoing in patients with MDS, AML, and chronic myelomonocytic leukemia (NCT02841540). Since SF3B1 mutation is observed in 90% of patients with MDS, good efficacy without side toxicity by administration with H3B-8800 will be expected.

### 6.2 SRPK inhibitor

Among protein kinase inhibitors, the therapeutic potential of splicing-related kinases SRPKs and CLKs has gained attention. Two clinical trials (NCT04247256 and NCT04652206) are currently being conducted *via* oral administration of an SRPK inhibitor, SCO-101 (**Table 1**). SCO-101 inhibits both ATP-Binding Cassette (ABC) G2 efflux pumps and SRPK1, thereby showing antitumor potential in combination with docetaxel in triple-negative breast cancer cells (161). A phase II clinical study has been conducted in patients with 5-flurouracil, leucovorin, and irinotecan (FOLFIRI)-resistant metastatic colorectal cancer (NCT04247256). The objectives of this study are 1) to determine the maximum tolerated dose (MTD) in patients receiving FOLFIRI plus escalating doses of SCO-101 and 2) to assess the safety, toxicity, and efficacy of the combination of SCO-101 and FOLFIRI in patients receiving FOLFIRI and the MTD of SCO-101 (162). Another clinical trial is a phase Ib study in combination with gemcitabine and nab-paclitaxel for patients with pancreatic ductal adenocarcinoma to establish the safety profile and MTD of SCO-101 (NCT04652206). Current clinical trials are focusing on tumors resistant to chemotherapy, because SCO-101 has the potential to suppress drug efflux *via* ABCG2. Additional remarkable profile for SCO-101 is SRPK1 inhibition. Cancer cell growth inhibition is proven in pancreatic cell lines by knockdown of SRPK1 through modulating alternative splicing, and upregulation of SRSF1 in pancreatic cancer is observed in patients resistant to gemcitabine (87, 163). Since preclinical studies have shown that SRPK1 regulates multiple splicing, a pharmacodynamic (PD) biomarker to monitor splicing alteration in patients should be developed.

**Table 1:** Small-molecule splicing modulators in clinical trial.

Molecular target	Agent name	Indications	Phase	Status	Clinical trial
SF3b	H3B-8800	MDS, AML, CML	I	Active, recruiting	NCT02841540
SRPK1	SCO-101	Metastatic colorectal cancer Metastatic pancreatic adenocarcinoma	I, II I, II	Active, recruiting Active, recruiting	NCT04247256 NCT04652206
CLK1-4	CTX-712	Advanced/relapsed/refractory malignant cancer Relapsed/refractory AML, high risk MDS	I I, II	Active, recruiting Active, not recruiting	JapicCTI-184188 NCT05732103
CLK1-4	SM08502	Advanced solid tumors	I I	Active, not recruiting Active, not recruiting	NCT03355066 NCT05084859
CDK12	THZ531	Ovarian carcinoma	II	Active, recruiting	NCT04555473
CDK7	SY-1365	Advanced solid tumors	I	Terminated	NCT03134638
CDK7	ICEC0942 (CT7001)	Advanced solid tumors	I, II	Completed	NCT03363893
CDK7	SY-5609	Advanced solid tumors	I	Active, not recruiting	NCT04247126
CDK7	LY3405105	Advanced solid tumors	I	Terminated	NCT03770494
PRMT5	GSK3326595	Advanced solid tumors Breast cancer Refractory MDS, CMML, AML	I II I	Active, not recruiting Completed Terminated	NCT02783300 NCT04676516 NCT03614728
PRMT5	PF06939999	Advanced/metastatic solid tumors	I	Terminated	NCT03854227
PRMT5	JNJ-64619178	Advanced solid tumors, NHL, and lower risk MDS	I	Active, not recruiting	NCT03573310
PRMT5	AMG 193	Advanced MTAP-null solid tumors	I, II	Active, recruiting	NCT05094336
PRMT5	PRT543	Advanced solid tumors and hematologic malignancies	I	Completed	NCT03886831
PRMT5	PRT811	Advanced solid tumors and recurrent gliomas	I	Completed	NCT04089449
PRMT5	SCR-6920	Advanced solid tumor and relapsed/refractory NHL	I	Active, recruiting	NCT05528055
PRMT5	TNG908	MTAP-deleted solid tumors	I, II	Active, recruiting	NCT05275478
Type I PRMT	GSK3368715	Solid Tumors and Diffuse Large B-cell Lymphoma (DLBCL)	I	Terminated	NCT03666988

The database of clinical trial was searched at ClinicalTrials.gov and Japic Clinical Trials Information on April 28, 2023. MDS, Myelodysplastic syndromes; AML, Acute myeloid leukemia; CML, Chronic myelomonocytic leukemia; CMML, Chronic myelomonocytic leukemia; NHL, Non-Hodgkin lymphoma; DLBCL, Diffuse large B-cell lymphoma.

### 6.3 CLK inhibitor

Two orally available CLK inhibitors (SM08502 and CTX-712) have entered clinical trials (**Table 1**). SM08502 is selective to CLK2 and CLK3 inhibition, thereby inhibiting phosphorylation of SR proteins (SRSF6) and enlarged nuclear speckle (164). In addition, SM08502 exerts antitumor activity in colorectal cancer cells *in vitro* and *in vivo* by decreasing gene expression and altering genes of the Wnt pathway, such as splicing of *LEF1*, *TCF7L2*, and *DVL1* (164). Currently, a phase I clinical trial is being conducted to evaluate the safety, tolerability, pharmacokinetics, PD, and preliminary antitumor efficacy of SM08502 in patients with advanced solid tumors resistant to standard therapy (NCT03355066), and another phase I study is being conducted in combination with hormonal therapy or chemotherapy in patients with castration-resistant prostate cancer, non-small cell lung cancer, and colorectal cancer (NCT05084859). CTX-712 is being orally administrated twice a week in a phase I study to determine the recommended dose (RD) by evaluating MTD, dose limiting toxicity, safety, pharmacokinetics, and PD profiles (JapicCTI-184188). Interim results from the phase I clinical trial revealed that two partial responses (PRs) and two complete responses (CRs) were observed in patients with ovarian cancer and AML, respectively, with a dose-dependent increase in splicing alteration (165). An additional interim phase I trial showed that four CRs and one CR with incomplete hematologic recovery were observed in eight patients with AML and MDS (166). Further phase I studies to determine the phase II dose will be started in patients with relapsed/refractory AML and higher risk MDS (NCT05732103), and detailed reports are expected to provide insightful information for cancer therapy.

### 6.4 CDK inhibitors

A phase II/III clinical study (NCT01580228) has been conducted using a pan-CDK (CDK1/2/5/9) inhibitor, dinaciclib, in patients with relapsed/refractory CLL (167). Administration of dinaciclib at escalating doses of 7 to 10 to 14 mg/m^2^ (on days 1, 8, and 15, respectively) in cycle 1 and 14 mg/m^2^ in cycle 2 and thereafter (1 cycle, 5, 28 days) for 12 cycles provides an acceptable safety and tolerability profile with typical CDK inhibitor-oriented adverse events represented by tumor lysis syndrome; however, its efficacy was limited and clinical trials were terminated (167). Encouraging preclinical data show that dinaciclib is also a potent inhibitor of CDK12 and sensitizes to triple-negative breast cancer cells in combination with PARP inhibitors by disrupting residual homologous recombination activity (168). THZ1, a covalent CDK7/12/13 inhibitor, exerts antitumor activity through transcriptional regulation by inhibiting CDK7 in acute T cell leukemia, MYCN-amplified neuroblastoma, small cell lung cancer, and triple-negative breast cancer (169–172). In addition to CDK7 inhibition, THZ1 and THZ531, CDK12/13 inhibitors, led to the reduction of the expression of DDR genes and induction of synthetic lethality, along with PARP inhibitors, in Ewing sarcoma cells *in vitro* and PDX model *in vivo* (173). THZ531 is currently being used in a longitudinal observational phase II study to assess its sensitivity *via* CDK12 inhibition using organoids derived from patients with high-grade serous ovarian cancer receiving PARP inhibitors (NCT04555473) (11). Most CDK12 inhibitors are still being studied in the preclinical stage, and several CDK12 selective inhibitors have shown promising results in the preclinical stage (174, 175). Since THZ1 is a substrate of multidrug transporters ABCB1 and ABCG2, tumor cells show resistance to THZ1. To overcome the resistance of THZ1, E9 was synthesized and overcame ABC-mediated resistance (176). Additionally, new types of CDK12 inhibitors as bifunctional protein degraders have emerged (177). Molecular glue compounds can mediate protein–protein interactions between a target protein, such as CDK12 and ubiquitin ligase to induce selective protein degradation (178). Proteolysis-targeting chimeras (PROTACs) consist of three elements: a ligand for the target protein, such as CDK12, a ligand for E3 ligase, and a linker to induce proteasome-mediated degradation of the target protein (178). Molecular glues and PROTACs increase selectivity for the target protein, such as CDK12, without inhibition of CDK13 function, although the compounds are still in the pre-clinical stage. In addition to CDK12, CDK7 inhibitor sensitizes DDR-proficient cancer cells to PARP inhibitor (179). SY-1365, a selective CDK7 inhibitor, inhibited cell growth in many different cancer types at nanomolar concentrations (180). In addition, SY-1365 decreased the level of oncogenic transcripts and DDR genes, such as *RAD51* and *CHEK1*. SY-1365 is currently under clinical trials for patients with ovarian and breast cancer (NCT03134638). Other three CDK7 selective inhibitors, namely, ICEC0942 (CT7001), SY-5609, and LY3405105, have progressed to phase I/II clinical trials (111), NCT03363893; NCT04247126; and NCT03770494). Multiple CDK9 inhibitors have also proceeded to clinical trials for patients with multiple solid and bladder tumors (181–183).

### 6.5 PRMT1 and PRMT5 inhibitors

Multiple PRMT5 inhibitors, which have potential antitumor activity and regulate splicing, are under clinical trials. An orally available PRMT5 inhibitor (GSK3326595) inhibited the methylation of Sm proteins and induced cell death in MCL *in vitro* and *in vivo* (147). Three clinical trials have been conducted in patients with solid tumors (NCT5094336), relapsed/refractory myelodysplastic syndrome (MDS), chronic myelomonocytic leukemia (CMML), hypoproliferative AML (NCT03614728), solid tumors, and non-Hodgkin’s lymphoma (NCT02783300). The adverse effects observed among the abovementioned trials were common, such as fatigue and anemia, but manageable, and PRs were observed in patients with several tumor types, such as human papillomavirus+ cervical cancer (1/1 subject) and ACC (3/14 subjects) in the NCT02783300 study (184). Clinical trials for other PRMT5 inhibitors are in progress: AMG 193, JNJ-64619178, PF-06939999, PRT543, PRT811, SCR-6920, and TNG908 (185), NCT05094336, NCT03573310, NCT03854227, NCT03886831, NCT04089449, NCT05528055, and NCT05275478), although it has not been reported that these inhibitors have splicing modulation activity.

To date, a limited number of PRMT1 inhibitors have proceeded to clinical trial. GSK3368715, a PRMT1 inhibitor, exerts antitumor activity *in vitro* and *in vivo* in multiple cancer types with splicing alteration activity of exon usage (186). A phase I clinical trial of GSK3368715 has been conducted in patients with relapsed/refractory diffuse large B-cell lymphoma and solid tumors. However, according to NCT03666988 of ClinicalTrials.gov, the current status of patient recruitment is terminated because overall benefit-risk profile did not support continuation of the study. Regarding patient stratification, co-deletion with methylthioadenosine phosphorylase (MTAP) and the tumor suppressor gene *CDKN2A* (p16) are observed in 40% of glioblastomas, 25% of melanomas and pancreatic adenocarcinomas, and 15% of non-small cell lung carcinomas (82). Subsequently, MTAP deficiency leads to the accumulation of 2-methylthioadenosine (MTA), which has the potential to inhibit PRMT5 activity (187–189). Genetic depletion of PRMT5 leads to vulnerability in MTAP deleted cells; however, GSK3235025, a PRMT5 inhibitor, surprisingly did not recapitulate vulnerability to PRMT5 depletion in MTAP deleted cells, implying that different inhibitory modes exist between MTA and small-molecule inhibitors, S-adenosylmethionine competitive and uncompetitive, respectively (188). Since the combination of PRMT1 and PRMT5 inhibitors exerts a synergistic antitumor effect (186), MTAP deficiency is associated with decreased induction of MMA and SDMA upon inhibition of type I PRMT activity. Given that the substantial population with MTAP deficiency includes many tumor types with limited therapeutic options, inhibition of type I PRMT activity by GSK3368715 may represent a promising approach for tumors of high unmet medical need with a defined patient selection strategy.

## 7 Future perspective and conclusions

Recent progress in multiple clinical trials targeting pre-mRNA splicing indicates that various splicing modulators have potential value as a novel class of antitumor agents. In this review, specific splicing-related kinases and related inhibitors entering clinical trials are described. It is one of the critical factors to consider patient stratification strategy for increasing the success rate in clinical trials. As described in this review, splicing factor-mutated tumors have recently been observed and attracted considerable attention, although the detailed mechanisms underlying splicing dysregulation in cancer remain unclear. Some of the SF3b complex inhibitors, such as H3B-8800, have the potential to be preferentially effective to splicing factor-mutated tumors compared to nonmutated tumors (**Table 2**). In addition, recent evidence has indicated that PRMT inhibitors also have potential for possibility of patient stratification for tumors with mutation of splicing factors since PRMT inhibitors of class I (PRMT1) or class II (PRMT5) preferentially inhibits cell viability and delayed disease progression in splicing factor, SRSF2^P95H^, SF3B1^Y765C^, or SF3B1^K700E^ mutated cells *in vitro* leukemia cells and *in vivo* AML PDX model compared to wild type cells (190) (**Table 2**). The results of ongoing clinical trial will be expected, and detailed molecular mechanism should be clarified to consider biomarker strategy. Other potential possibility for patient stratification is SRPK inhibitor, which specifically showed antitumor efficacy in AML cells since alternative splicing of BRD4 was altered by SRPK inhibitor (90). The inhibitors targeting bromo- and extra-terminal domain (BET) including BRD4 have remarkable antitumor activity in preclinical studies, and more than 20 clinical trials have been completed and ongoing (191). To date, limited clinical efficacies for BET inhibitor have been observed implying that predictive biomarker, pharmacodynamics marker, and combination strategy is lacking in clinical trials. Since SRPK inhibitors modulate multiple splicing events involving aberrantly expressed oncogenes, it is conceivable that SRPK inhibitor is more effective compared to BET inhibitor through combination of antitumor effect by multiple splicing changes. In addition, BRD4 forms fusion proteins with nuclear protein in testis (NUT). The BRD4-NUT fusion proteins have aggressive oncogenic property and involve in NUT-midline carcinoma (NMC) with a poor prognosis (192). In addition to AML, SRPK inhibitor could be effective to NMC, though toxicity should be carefully validated and monitored.

**Table 2:** Potential patient stratification and drug combination strategy in clinical.

Inhibitor	Cancer types	Genetics	Drug combination
SF3b	MDS, CLL, AML, solid tumors	Mutation in splicing factors (eg. SF3b)	Bcl-2/Bcl-xL inhibitor
SRPK1	AML	ND	ND
CLK1-4	ND	Amplification in Myc or CLK2	Bcl-2/Bcl-xL inhibitor
CDK12	Ovarian, breast, prostate cancer	BRCA wildtype	PARP inhibitor
CDK12	ND	ND	CDK7 inhibitor
PRMT5	MDS, CLL, AML, solid tumors	Mutation in splicing factors (eg. SF3b)	PRMT class I inhibitor
PRMT5	Solid tumors	MTAP deletion	PRMT class I inhibitor

ND, Not determined; MDS, Myelodysplastic syndromes; CLL, Chronic lymphocytic leukemia; AML, Acute myeloid leukemia.

Additional potential possibility of patient stratification suggests that, in MYC-driven cancer cells, increase of total RNA synthesis and protein translation lead to increased burden on the core spliceosome (193). This evidence supports that CLK inhibitor (T-025) sensitives to MYC-amplified tumors as well as CLK2 upregulated tumors (103). The AACR Project GENIE, an international data-sharing consortium, showing clinical-grade cancer genomic data with clinical outcome data for tens of thousands of cancer patients revealed that the amplification of MYC is predominantly found in breast invasive ductal carcinoma, lung adenocarcinoma, colon adenocarcinoma, prostate adenocarcinoma, and invasive breast carcinoma (194). Independent analysis based on Cancer Genome Atlas using 489 high-grade serous ovarian adenocarcinomas (HGSOC) showed that chromosome 8q including MYC has the most significant gains and occurred in 65% of HGSOC (195), and MYC amplification was the highest frequency in ovarian cancer compared to other tumor types (196). Additionally, MYC transcription is reduced by THZ531, a CDK12/13 inhibitor *in vitro* and *in vivo*, patient-derived xenografts from ovarian cancer patients, not CDK7 inhibitor (196). These evidences suggest that MYC dependent tumors, such as HGSOC, could be effective for CLK inhibitor and CDK12/13 inhibitor (**Table 2**).

In addition to patient stratification strategy, it is of particular importance to consider molecular mechanism-based combination therapy strategy. Independent group shows that SF3b inhibitor, E7107, or CLK inhibitor (T3) alters splicing of anti-apoptotic *MCL1*, but not that of anti-apoptotic Bcl-2 family, such as Bcl-2 and Bcl-xL (**Figure 4**) (197, 198). Combination of E7107 or T3 with Bcl-xL/Bcl-2 inhibitor synergistically induced apoptosis in cancer cells. Therefore, drug combination strategies with splicing modulator and Bcl-xL/Bcl-2 inhibitors could be effective. However, more detailed analysis should be needed to clarify the reason why splicing inhibitors are insensitive to splicing of Bcl-xL and Bcl-2, and identify the predictive biomarkers in clinics.

CDK12 inhibitor could be ideal to induce synthetic lethal in combination with PARP inhibitor in BRCA wildtype of tumor patients. Although CDK7 inhibitor also sensitizes DDR-proficient gene to PARP inhibitor, it has been suggested that the molecular mechanism between CDK12 and CDK7 would be different. Whereas CDK7 involves in oncogene-related transcription and alternative splicing, CDK12 regulates alternative premature intronic polyadenylation. This evidence implies that combination with CDK12 inhibitor and CDK7 inhibitor has synergistic antitumor effect, in particular, in ovarian and breast DDR-proficient tumors. PRMT inhibitors also have potential for possibility of drug combination. Combination of PRMT1 inhibitor and PRMT5 inhibitor shows stronger synergistic cell growth effect and antitumor efficacy *in vitro* and *in vivo* splicing factor-mutated cells compared to wild type cells (190). To date, the number of CDK12 selective inhibitor or PRMT1 inhibitor is limited in clinical trial. In addition to ATP-competitive inhibitors, molecular glue and PROTACs technology have recently gained attention to obtain selective CDK12 inhibitors due to the high homology between kinase domains of CDK12 and other CDKs, especially CDK13 (134, 199–202). It is highly expected that more selective CDK12 inhibitors will proceed to clinical trials to show antitumor effect through precise molecular mechanism.

Further validation of the accuracy of patient stratification in a clinical trial is awaited and will pave the way to cure cancers through modulating pre-mRNA splicing. To achieve these, molecular machinery modulating alternative splicing should be clarified more precisely *via* development of new type of selective compounds, such as molecular glues and PROTACs, and modalities targeting pre-mRNA splicing-related molecules. The evidence will lead to define patient stratification and combination therapy strategy for patients with cancer in near future.

## Author contributions

SA conceived and designed the overall manuscript. SA, MO, and MY wrote the manuscript. All authors contributed to the article and approved the submitted version.
